# A cholecystectomy revealing sarcoidosis: A case report

**DOI:** 10.1016/j.ijscr.2025.111114

**Published:** 2025-03-03

**Authors:** Sarra Ben Rejeb, Mariem Ayari, Soumaya Debbiche, Hela Cherif, Mehdi Charfi, Taieb Jomni

**Affiliations:** a Pathology Department, Security Forces Hospital, Marsa, Tunisia; b Gastrology department, Security Forces Hospital, Marsa, Tunisia; c Pneumology department, Security Forces Hospital, Marsa, Tunisia; d Radiology department, Security Forces Hospital, Marsa, Tunisia

**Keywords:** Sarcoidosis, Biliary, Lymph node, Gallbladder

## Abstract

**Introduction and importance:**

Sarcoidosis is a multisystemic, non-caseating granulomatous disease of unknown etiology, predominantly affecting the lungs and lymph nodes. Gastrointestinal involvement, especially in the gallbladder, is rare. Here, we presented a unique case of granulomatous lymphadenitis in a gallbladder-associated lymph node, revealing systemic sarcoidosis.

**Case presentation:**

A 70-year-old woman with a history of atrial fibrillation, hypertension, and autoimmune gastritis presented with right upper quadrant pain and vomiting. Ultrasound showed multiple gallstones, and laboratory tests revealed elevated liver enzymes. Cholecystectomy was performed, and histopathological examination of the gallbladder specimen revealed granulomas suggestive of sarcoidosis. The patient had a two-month history of dyspnea, cough, and sputum production. Radiologic imaging revealed interstitial lung disease and mediastinal lymphadenopathy, supporting a diagnosis of sarcoidosis. Laboratory tests confirmed elevated ACE levels, and tuberculosis was ruled out.

**Clinical discussion:**

Sarcoidosis is often asymptomatic or misdiagnosed due to its variable presentations. Involvement of the gallbladder or biliary lymph nodes is exceedingly rare, typically discovered incidentally during cholecystectomy. Histological findings of non-caseating granulomas with asteroid and Schaumann bodies are indicative of sarcoidosis. In endemic regions, tuberculosis must be excluded. Clinical reassessment is crucial to detect symptoms of sarcoidosis following incidental findings.

**Conclusion:**

This rare presentation of sarcoidosis in a biliary lymph node highlights the importance of thorough pathological examination in cases of gallbladder abnormalities with lymph node involvement. A non-caseating granulomas should raise suspicion of sarcoîdosis even in asymptomatic patients.

## Highlights

• This case report described an unusual presentation of sarcoidosis involving a gallbladder-associated lymph node, an extremely rare site for the disease.
• The case underscored the importance of histopathological examination in identifying non-caseating granulomas and highlighted the need for multidisiplinary reassessment to confirm systemic sarcoidosis.
• Sarcoidosis should always be considered in non caseiting granulomatous lymphadenitis

## 1 Introduction

Sarcoidosis is a multisystemic non-caseating granulomatous disease of unknown etiology that may affect all organs at various rates, but has a wide predilection for the lungs and lymph nodes [1,2]. Since the lungs and lymph nodes are the most commonly involved sites, patients mainly present with mediastinal lymph node enlargement or pulmonary complaints such as shortness of breath or dry cough [[1], [2], [3]]. However, in cases of extra-pulmonary and lymph node involvement, patients may be either asymptomatic or have nonspecific complaints. In these cases, the diagnosis of sarcoidosis may not be considered, leading to a diagnostic and therapeutic delay [2,3]. Among the atypical sites of sarcoidosis, the gastrointestinal involvement is extremely rare with less than 4 % of sarcoidosis having GI and hepatic lesions with the stomach and small intestine being the most involved [4,5].

Gallbladder involvement in sarcoidosis is even rare, with only a few reported cases in the literature [3,[6], [7], [8], [9]]. Most of these cases are incidentally discovered during pathological examination of gallbladder specimens revealing the disease [6].

We herein reported a rare case of granulomatous lymphadenitis of the gallbladder revealing a systemic sarcoidosis.

## 2 Case presentation

**Fig. 1:**
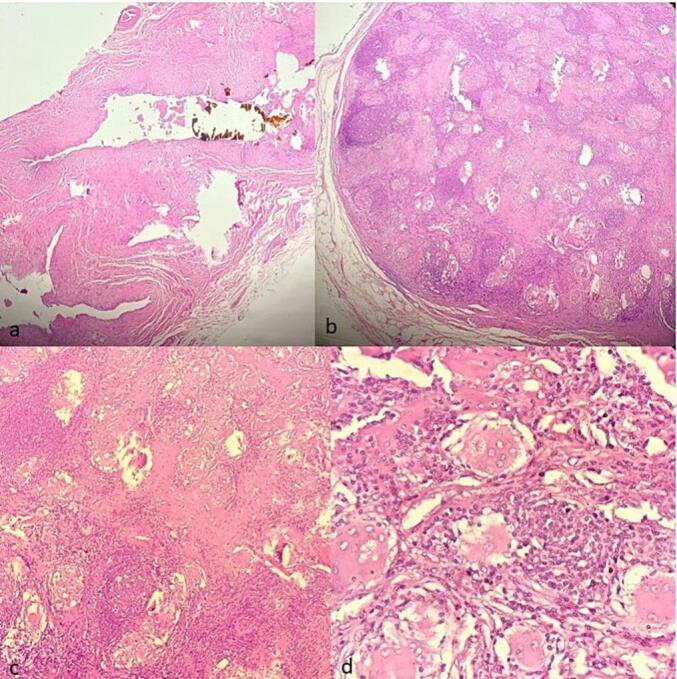
Pathological findings: (a) HE X 4 gallbladder wall showing features of chronic cholecystitis with lumen lithiasis (b) HE x 4: cystic duct lymph node with multiple non caseating granulomas (c) HE X 20: the granulomas are composed of epithelioid and giant cells surrounded with fibrosis (d) HE X 40: intra-cytoplasmic “asteroîd bodies” within a giant cell.

**Fig. 2:**
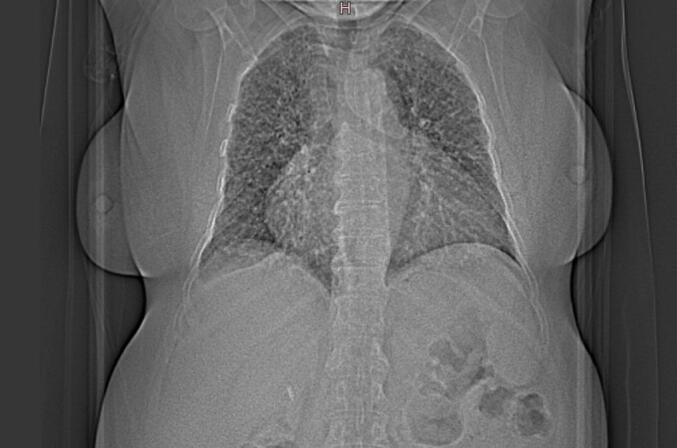
Chest radiography showing bilateral interstitial lung disease.

**Fig. 3:**
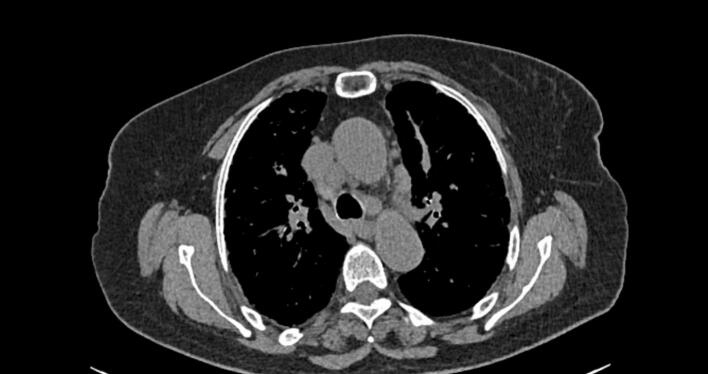
Computed tomography of the thorax revealing a diffuse interstitial pneumonia pattern with mediastinal lymphadenopathies.

This work has been reported in accordance with the SCARE criteria [10]. A 70 year-old woman with medical history of atrial fibrillation, high blood pressure and auto-immune gastritis presented to the emergency department for right upper quadrant pain associated with vomiting. Abdominal ultrasound revealed multiple stones in the gallbladder lumen. On laboratory tests, the patient had high levels AST(95UI/L), ALT(75UI/L) and GGT(76UI/L). The patient underwent cholecystectomy. On gross examination, the submitted specimen measured 9 cm. It has a thickened wall with gallstones in the lumen. A 1.5 cm lymph node was identified at the neck region. Microscopic examination revealed signs of chronic cholecystitis. The cystic lymph node showed numerous non-necrotizing epithelioid and multinucleated giant cell granulomas. These granulomas were small, non-confluent, and surrounded by collagenous fibrosis. The giant cells contained eosinophilic intracytoplasmic inclusions with a stellate appearance, suggestive of “asteroid bodies”, as well as basophilic inclusions possibly corresponding to foreign bodies or “Schaumann bodies” (Fig. 1). Those findings were highly suggestive of sarcoidosis. Postoperative, the patient was referred to the pulmonary disease department for further explorations. During the clinical interview, the patient reported a two-month history of exertional dyspnea accompanied by cough and whitish sputum. A chest radiograph demonstrated features of bilateral interstitial lung disease (Fig. 2). Computed tomography of the thorax revealed a diffuse interstitial pneumonia pattern with mediastinal lymphadenopathy, raising suspicion for sarcoidosis (Fig. 3). Oln laboratory tests, increased levels of angiotensin-converting enzyme (ACE) were found. Calcium level was normal. There were no clinical or biological signs suggestive of tuberculosis, particularly in the absence of tuberculous contact, fever, or night sweats. The tuberculin skin test (TST) was negative, and the search for acid-fast bacilli (AFB) in the bronchial fluid was also negative. Similarly, hematological disorders were ruled out due to the absence of general condition deterioration, weight loss, peripheral lymphadenopathy, and a normal lactate dehydrogenase (LDH) level. Based on these findings, the diagnosis of systemic sarcoidosis was made.

## 3 Discussion

Sarcoidosis is a rare a multi-system disease of unknown etiology characterized by the infiltration of various organs by non-necrotizing granulomas [1,11]. Although sarcoidosis can affect multiple organs, nearly half of patients remain asymptomatic, which often leads to a delayed diagnosis [3]. The diagnosis of sarcoidosis relies on three key criteria: a sufficiently characteristic clinical presentation, histological findings of non-caseating granulomas in one or more tissue samples, and the exclusion of alternative other causes of granulomatous diseases [12]. Hence, sarcoidosis should be considered when noncaseating granulomas are found in biopsies, even in the absence of clinical symptoms [3]. However, the initial diagnosis of sarcoidosis with gallbladder and/or gallbladder-associated lymph node involvement is very rare with only few reported cases in the literature [9]. In this paper, we described a rare case of granulomatous lymphadenitis in a gallbladder-associated lymph node, revealing a previously unrecognized sarcoidosis.

Clinically, most patients with gallbladder involvement in sarcoidosis either presented with symptoms mimicking acute cholecystitis or remained asymptomatic and as in our case, the diagnosis was often made incidentally during cholecystectomy [6,9]. In other cases, the symptoms may mimic those of an infectious etiology, which must be considered in the differential diagnosis [9]. Inflammation affecting the biliary tract and lymph nodes can also lead to extrinsic compression of the cystic duct, resulting in increased jaundice. In addition, in rare cases, the strictures in the extrahepatic ducts resulting from biliary ducts sarcoidosis may be confused with a malignant process [6,9,13,14].

Regardless of the clinical presentation, the incidental finding of non-caseating granulomas in the gallbladder or biliary lymph node during routine pathological examination of chronic cholecystitis should raise suspicion of sarcoidosis [3]. As in this described case, these granulomas are typically composed of tightly clustered epithelioid cells with some multinucleated giant cells and few lymphocytes and are often surrounded by fibrosis. The finding of asteroid and Schaumann bodies in giant cells, although not specific, is highly suggestive of sarcoîdosis [12]. In the absence of a known sarcoidosis diagnosis, it is necessary to rule out other causes of non caseating granulomatosis [6]. In particular, in endemic countries such as ours, tuberculosis should be actively investigated. In this reported case, all investigations were performed to rule out tuberculosis; TST was negative, and the search for AFB in the bronchial fluid was also negative. Other less common conditions should also be considered such as a sarcoid-like reaction in metastatic lymph nodes [14].

In cases of incidental discovery, as in our presentation, a clinical reassessment of the patient is necessary to identify any warning signs suggestive of sarcoidosis. Indeed, in our patient, pulmonary symptoms characterized by dyspnea, cough, and sputum production were identified. In radiological explorations, we also found a diffuse interstitial pneumonia pattern with mediastinal lymphadenopathy, consistent with sarcoidosis. Finally, on laboratory tests, increased levels of ACE were also found. A final diagnosis of sarcoidosis was made based on clinical, radiological, biological and pathological findings. Although the treatment of systemic sarcoîdosis is mainly based on steroid therapy, there is limited data regarding the specific treatment of gallbladder sarcoîdosis [6]. However, in most published cases, cholecystectomy was the mainstay of treatment [3,7,9,14].

In this reported case, considering the multi-organ involvement, the patient received prednisone.

## 4 Conclusion

This rare presentation of sarcoidosis in a biliary lymph node highlights the importance of thorough pathological examination in cases of gallbladder abnormalities with lymph node involvement. A non-caseating granulomas should raise suspicion of sarcoîdosis even in asymptomatic patients. A clinical re-assessment may reveal symptoms related to sarcoîdosis. Ultimately, we only find what we actively look for.

## CRediT authorship contribution statement

All the authors read and approved the final version of the manuscript.

Sarra Ben Rejeb (MD): conception, acquisition of data, literature research and preparing the manuscript.

Mariem Ayari (MD): conception, literature research supervision and revising the manuscript.

Soumaya Debbiche (MD): Imaging data acquisition, revising and editing

Hela Cherif (MD): Imaging data acquisition, revising and editing

Mehdi Charfi (Pr): radiological imaging analysis and acquisition

Taieb Jomni (Pr) Supervision and revision

## Consent for publication

Written informed consent for publication of patient's clinical details and clinical images was obtained from the patient.

## Ethics statement

The ethics approval is not required for case reports deemed not to constitute research at my institution.

## Guarantor

Dr. Sarra Ben Rejeb

## Sources of funding

None.

## Declaration of competing interest

None.
